# Diameters of the normal thoracic aorta measured by cardiovascular magnetic resonance imaging; correlation with gender, body surface area and body mass index

**DOI:** 10.1186/1532-429X-15-S1-E77

**Published:** 2013-01-30

**Authors:** Anne Davis, Cameron Holloway, Adam J Lewandowski, Ntobeko Ntusi, Richard M Nethononda, Alex Pitcher, Jane M Francis, Paul Leeson, Stefan Neubauer, Oliver J Rider

**Affiliations:** 1 Oxford Centre for Magnetic Resonance Research, University of Oxford, Oxford, UK; 2 Oxford Clinical Cardiovascular Research Facility, University of Oxford, Oxford, UK

## Background

Although it is widely accepted that obesity is related to increased aortic size, to date, there is no large study assessing the effect of obesity, in the absence of co-morbidities, on regional aortic diameter. In addition, despite the fact that magnetic resonance imaging (MRI) is generally regarded as the gold standard technique for imaging the aorta, the vast majority of data comes from 2D echo, which has marked limitations in the setting of obesity. As a result, we aimed to use MRI to 1) establish a large gender specific normal database of reference diameters for the aorta and 2) investigate the effect of increasing body surface area (BSA) on aortic size.

## Methods

484 subjects (230 male, 254 female), age 19-70 years without identifiable cardiac risk factors (BMI range 15.7 - 52.6) underwent MRI to determine aortic diameter at three levels namely; the ascending aorta (Ao) and proximal descending aorta (PDA, pulmonary artery level), and the abdominal aorta (DDA, 12 cm below the PDA level). In addition, 208 of the subjects had aortic root measurements performed at the aortic valve annulus, aortic sinuses and sino-tubular junction.

## Results

**Figure 1:**
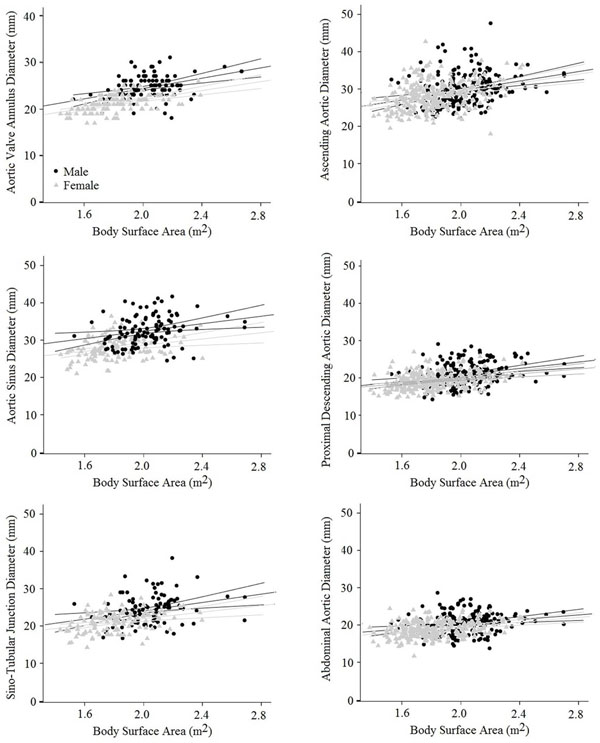
The effect of gender on the relationship between regional aortic diameter and body surface area.

All subjects were normotensive (SBP 121±12, DBP 75±9 mmHg), normoglycaemic (4.8±0.5 mmol/l) and normocholesterolaemic (4.9±0.8 mmol/l) on the day of scanning. As expected, with increasing BSA, aortic root diameter increased at all levels measured (AV annulus ♂+5.5, ♀+4.4 mm, Aortic Sinus ♂+5.1, ♀+4.2 mm, ST-junction ♂+5.7, ♀+4.4 mm all per m^2^ (BSA) increase, Table 1, Figure 1). No gender difference in the degree of dilatation with increasing BSA was seen (p>0.5). Aortic diameters at the more distal aortic levels also increased with increasing BSA (Ao, ♂+6.5, ♀+6.1 mm, PDA ♂+4.4, +♀3.4, DDA ♂+3.2, ♀+3.3 mm, all per m^2 ^BSA increase, Figure 1). Again, no gender differences in the degree of dilatation were seen (p>0.5 for all analyses). However, the degree of regional aortic dilatation in both male and female obesity without co-morbidities was minor (AV annulus ♂+0.17, ♀+0.08 mm, ST junction ♂+0.16, ♀ +0.19 mm, Ao ♂+0.21, ♀+0.18 mm, PDA ♂+0.16, ♀ +0.09 mm, DDA ♂+0.15, ♀+0.09 mm, per BMI point increase, p<0.05 for all analyses).

**Table 1:** Gender Specific Effects of Obesity on Regional Aortic Diameter - Data Presented as Mean With Normal Range (+/- 2SD)

		Male		
Aortic Diameter (mm)	Normal Weight	Overweight	Obese	ANOVA p
Aortic Valve Annulus	24.0 (18.8-29.2)	24.7 (19.5-29.9)	25.7 (20.7-30.7)	<0.05
Sinus of Valsalva	32.2 (24.6-39.8)	32.9 (25.3-40.5)	33.3 (25.3-31.3)	<0.05
Sino-tubular Junction	24.9 (18.1-31.7)	25.8 (17.0-34.6)	25.9 (19.1-32.7)	<0.05
Ascending Aorta	26.6 (18.2-35.0)	27.8 (18.8-36.8	28.6 (23.2-34.0)	<0.01
Proximal Descending Aorta	20.4 (14.6-26.2)	21.2 (15.6-26.8)	22.1 (16.5-27.7)	<0.01
Distal Descending Aorta	17.4 (12.0-22.8)	18.3 (12.7-23.9)	19.0 (14.8-23.2)	<0.01
BMI (kg/m2)	22 +/-1.7	27 +/-1.6	34 +/-4.8	<0.01
BSA (m2)	1.9 (+/-0.1)	2.0 (+/-0.1)	2.3 (+/-0.2)	<0.01

		Female		

Aortic Diameter (mm	Normal Weight	Overweight	Obese	ANOVA p
Aortic Valve Annulus	20.2 (17.0-23.4)	21.7 (18.5-23.9)	21.6 (17.6-25.6)	<0.01
Sinus of Valsalva	27.6 (22.0-33.2)	28.6 (21.6-35.6)	27.8 (22.2-33.4)	<0.05
Sino-tubular Junction	21.7 (16.7-26.7)	22.5 (16.5-28.5)	22.3 (16.5-28.1)	<0.05
Ascending Aorta	24.8 (17.6-32.0)	26.7 (19.3-34.1)	26.9 (19.3-34.5)	<0.01
Proximal Descending Aorta	18.6 (14.6-22.6)	19.5 (14.9-24.1)	20.1 (15.5-24.7)	<0.01
Distal Descending Aorta	16.1 (14.1-18.1)	16.9 (14.7-19.1)	17.6 (15.7-19.5)	<0.01
BMI (kg/m2)	22.0 (+/-1.6)	27.0 (+/-1.5)	37.0 (+/-4.8)	<0.01
BSA (m2)	1.7 (+/-0.1)	1.8 (+/-0.1)	2.0 (+/-0.2)	<0.01

## Conclusions

Aortic diameters were larger in males than females at all levels measured. Across both genders, obesity, in the absence of traditional cardiovascular risk factors, is characterized by a minor degree of aortic dilatation. There are no significant gender differences in the degree of dilatation with increasing obesity.

## Funding

The study was supported by grants from the British Heart Foundation and Wellcome Trust and by the Oxford Partnership Comprehensive Biomedical Research Centre with funding from the Department of Health's NIHR Biomedical Research Centres funding scheme. SN acknowledges support from the Oxford BHF Centre of Research Excellence

